# OCT for Optimizing Long-Term Clinical Results in Left Main PCI—Dream or Reality? Results from a Single-Center High-Volume Registry

**DOI:** 10.3390/jcm14165824

**Published:** 2025-08-18

**Authors:** Florin-Leontin Lazar, Teodor Paul Kacso, Calin Homorodean, Mihai Ober, Horea-Laurentiu Onea, Dan Tataru, Mihai Spinu, Maria Olinic, Minodora Teodoru, Dan-Mircea Olinic

**Affiliations:** 1 Medical Clinic Number 1, 4th Department of Internal Medicine, “Iuliu Haţieganu” University of Medicine and Pharmacy, 400023 Cluj-Napoca, Romania; lazar.leontin@yahoo.com (F.-L.L.); onea.lau@gmail.com (H.-L.O.); tataru.cardio@gmail.com (D.T.); spinu_mihai@yahoo.com (M.S.); maria.olinic@yahoo.com (M.O.); danolinic@gmail.com (D.-M.O.); 2 Sibiu County Emergency Hospital, 550245 Sibiu, Romania; minodora.teodoru@ulbsibiu.ro; 3 Department of Interventional Cardiology, Cluj County Emergency Hospital, 400006 Cluj-Napoca, Romania; teokacso@gmail.com (T.P.K.); mihai_ober@yahoo.com (M.O.)

**Keywords:** optical coherence tomography, left main, PCI, long-term mortality

## Abstract

**Background:** With growing evidence regarding long-term clinical results of left main angioplasty, it has become clear that the gap between percutaneous coronary interventions (PCIs) and bypass surgery can be narrowed only by improving the PCI technique. While intravascular ultrasound (IVUS) has become routinely used for this subset of lesions, there is still insufficient data regarding the role of optical coherence tomography (OCT) in left main PCI. **Aims:** The aim of this study was to investigate the long-term results of OCT-guided PCI in comparison to angiographical guidance alone. **Material and methods:** We conducted a retrospective single-center high-volume analysis of patients with left main disease treated by PCI. The primary endpoint was all-cause death. **Results and discussion:** Between January 2013 and January 2024, we enrolled 221 eligible patients with unprotected left main coronary artery disease treated by PCI; among these, 13.1% were treated by OCT-guided PCI and 86.9% by angiographic-guided PCI. At a median follow up of 30.16 months (interquartile range: 14.3–60 months), Kaplan–Meier survival analysis revealed a significantly higher survival probability in the OCT group compared to the non-OCT group (log-rank *p* = 0.034), with no significant differences between the groups regarding procedural success rate. In the multivariable Cox proportional hazards model, adjusting for other relevant covariates, OCT was borderline non-significantly independently associated with a 63% reduction in mortality (HR = 0.37, *p* = 0.063). **Conclusions:** In our study, OCT-guided PCI was associated with early procedural distinctions and a trend toward improved unadjusted survival in LM PCI. The findings highlight the potential procedural advantages of OCT, as well as the need for larger prospective studies to establish its long-term clinical benefits in left main interventions.

## 1. New Findings

Coronary artery disease represents the leading cause of mortality worldwide, with left main disease being one of the most challenging presentations of the disease. In this retrospective study on 221 patients, we observed that an intracoronary imaging technique (optic coherence tomography—OCT) may improve long-term survival for left main percutaneous coronary interventions (PCIs) when compared to angiographic guidance alone.

## 2. Introduction

Left main (LM) percutaneous coronary interventions (PCIs) still represent a subject of great debate in terms of long-term safety and efficacy as the results reported by the primary studies comparing this strategy to coronary artery bypass grafting (CABG) are still controversial [1,2].

The EXCEL trial, which included 1905 patients with LM disease, reported a similar death rate from any cause, stroke, or myocardial infarction (MI) between the PCI group (948 patients) and the CABG group (957 patients) (22.0% vs. 19.2%; difference: 2.8 percentage points; 95% confidence interval [CI]: −0.9 to 6.5; *p* = 0.13); however, a higher rate of death from any cause (13.0% vs. 9.9%; difference: 3.1 percentage points; 95% CI: 0.2 to 6.1) and a higher revascularization rate (16.9% vs. 10.0%; difference: 6.9 percentage points; 95% CI: 3.7 to 10.0) were reported in the PCI group [1]. On the other hand, despite important differences in terms of the definition of MI, patient assessment, procedural characteristics, device used, and methodology [3], the NOBLE trial [2] found CABG to be superior to PCI for the composite of all-cause mortality, non-procedural myocardial infarction, repeat revascularization, and stroke. Furthermore, even longer-term results are now available from the SYNTAX [4] and PRECOMBAT [5] trials, which did not find any significant differences at 10 years of follow-up between PCI and CABG in terms of all-cause death [4] or major adverse cardiac or cerebrovascular events (MACCEs) [5]. Recently, a meta-analysis of these major trials found no statistically significant difference in 5-year all-cause death between PCI and CABG [6].

As a result of all of these studies, a 2022 Joint European Society of Cardiology (ESC)/European Association for Cardio-Thoracic Surgery (EACTS) task force reviewed the 2018 guideline recommendations on the revascularization strategy in low-risk surgical patients with LM disease. According to this document, as well as being recently confirmed by the 2024 ESC guidelines on chronic coronary syndromes, PCI is now recommended as an alternative to CABG; however, an IIA class is recommended for using intravascular imaging in order to optimize LM PCI [7,8]. Multiple studies have demonstrated the benefits of intravascular ultrasound (IVUS) for guiding LM procedures; however, the role of OCT in this setting is still unclear due to its well-known limitations in large vessels and ostial lesions.

Regarding the benefits of IVUS, a sub-study of the above-mentioned NOBLE trial identified that 435 out of the 603 patients included in the study underwent IVUS guidance [9]. In this subset of patients, the rate of target lesion revascularization (TLR) was significantly reduced in the IVUS group (5.1% vs. 11.6%, *p* = 0.01); no influence was seen in relation to MACCE, death, myocardial infarction, or stent thrombosis at the 5-year follow-up [9]. More encouraging results were reported from the MAIN-COMPARE study, in which the 3-year mortality rate was lower with IVUS guidance as compared with angiography guidance [10], without the influence of target vessel revascularization or MI rates.

There are now several studies that have investigated the role of OCT in LM PCI. The LEMON trial enrolled 70 patients with LM disease, for which OCT was used according to a pre-specified protocol; the primary endpoint was procedural success, which was defined as residual angiographic stenosis < 50% + TIMI 3 flow in all branches + adequate OCT stent expansion [11]. With a change in the operator’s strategy in 26% of the cases and a 98.6% one-year survival free from MACE, the study demonstrated the feasibility of OCT-guided LM PCI. Another pivotal study was the ROCK II retrospective multicenter study, which compared the performance of two different imaging techniques, OCT and IVUS, and angiographic guidance in 730 patients with LM disease (162 OCT, 215 IVUS, 353 angiographic guidance) [12]. Target lesion failure (TLF) was the primary endpoint and at one-year follow-up, it was significantly reduced by intravascular imaging when compared to angiographic guidance (21.2% vs. 12.7%, *p* = 0.039), however without any differences between the two imaging modalities (*p* = 0.26) [12].

Taking into consideration that the mean follow-up of these studies was one year, the aim of our registry was to investigate the role of OCT in improving long-term results for LM PCI, as data is still scarce.

## 3. Materials and Methods

### 3.1. Study Design

We conducted a retrospective study in a high-volume center from Cluj-Napoca, Romania, with the aim of investigating the role of OCT in improving long-term hard clinical endpoints in left main disease treated by PCI. The inclusion criteria were (a) age > 18 years and informed consent; (b) significant left main disease (>50% stenosis) treated by PCI; and (c) at least one OCT run during the index procedure (OCT group). The exclusion criteria were (a) multiple system organ failure (MSOF); (b) cardiogenic shock (c) LVEF < 25%; and (d) contraindication to drug-eluting stent (DES) implantation.

A binary logistic regression model was used to explore potential predictors of OCT use. No significant associations were identified, indicating the absence of strong selection patterns based on the available covariates. As such, formal propensity score matching (PSM) was not pursued. However, to avoid extrapolating treatment effects to patients who would never have been considered for OCT, we applied one-sided trimming based on the region of common support derived from the logistic model predicting OCT use. Namely, only patients with a predicted probability of OCT use ≥ 0.0469 were enrolled (the minimum probability observed in the OCT group). This allowed us to limit the analysis to patients who could plausibly have received the intervention. To reduce bias due to differences in observation periods, follow-up was restricted to a maximum of 60 months.

“True” bifurcation lesions were defined as having significant (>50%) stenosis in both the main branch (MB) and the side branch (SB) (i.e., Medina 1.1.1; 1.0.1; or 0.1.1), whereas the rest were classified as “simple”. A separate category was assigned for LM ostial lesions irrespective of the distal aspect of the LM. Indication was classified as STEMI, NSTEMI, or elective coronary angiography (including unstable angina that did not require urgent (<24 h) coronary angiography). For the cases treated by angiographic guidance only, lesion length was obtained using the length of the DES or the DCB, or the sum if more than one device was used. The vessel diameter was obtained from the maximum diameter of the DCB or DES used, or the post-dilatation balloon, as 1:1 ratio to visual estimation of vessel diameter was mandatory. In the imaging-guided procedures, the length and diameter of the culprit vessel/vessels were dictated by the OCT findings. All bifurcation stenting techniques were allowed, including a hybrid approach using a DES for LM-LAD treatment and a DCB for ostial LCX treatment. As this was a retrospective analysis, the decision to use OCT was at the discretion of the operator at the time of the procedure. This study was approved by the ethic committee of our institution, and all the patients provided written informed consent.

### 3.2. Endpoints

The primary endpoint was defined as all-cause death at the longest available follow-up. The secondary endpoint was the procedural success rate, defined according to the LEMON criteria (residual angiographic stenosis < 50% + TIMI 3 flow in all branches + adequate OCT stent expansion). In the non-OCT group, procedural success was defined as residual angiographic stenosis < 50% + TIMI3 flow in all branches + good expansion of the final balloon used for the proximal optimization technique (POT) or the stent’s balloon, as visually assessed.

Follow-up was performed through ambulatory visits or telephone contact, while for the cases where we were unable to contact them, we obtained the survival registries from the local authorities.

Our study protocol was carried out according to the Declaration of Helsinki and was endorsed by the University of Medicine and the Pharmacy “Iuliu Hatieganu” Clinical Research Ethics Committee (AVZ 199/3, on 26 July 2023).

### 3.3. Statistical Analysis

Statistical analyses were carried out using SPSS version 30.0 in which comparisons between the OCT and non-OCT groups were performed using the Chi-square test for categorical variables and, for continuous variables, Student’s *t*-test for independent samples or Mann–Whitney U test in the case of non-parametric variables. Parametric variables are presented as mean ± S.D and non-parametric variables as median (IQ range).

Survival analysis was conducted using Kaplan–Meier estimates, and differences between groups were assessed with the log-rank (Mantel-Cox) test. To adjust for potential confounding factors, a multivariable Cox proportional hazards regression model was constructed. The proportional hazard (PH) assumption was assessed using log-minus-log survival plots and time-dependent covariate testing, created by multiplying the covariate by the natural logarithm of time. None of the covariate–time interaction terms were statistically significant (all *p* > 0.10), indicating no violation of the PH assumption.

Multicollinearity was assessed using Tolerance and Variance Inflation Factor (VIF) statistics; no significant multicollinearity was observed (all Tolerance values > 0.7 and VIF < 1.5).

## 4. Results

### 4.1. Baseline Clinical and Procedural Characteristics

**Figure 1:**
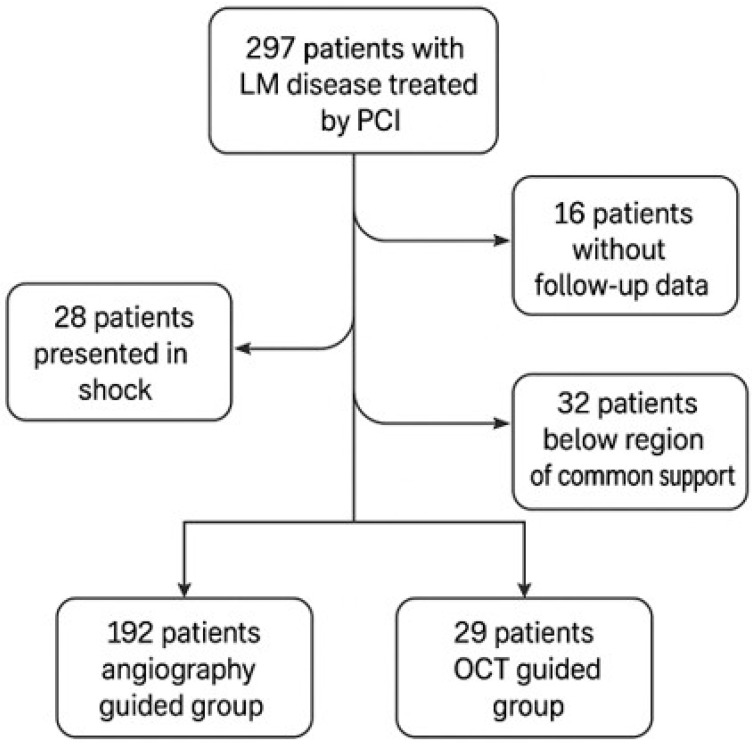
Flow-chart of patient selection. LVEF: left ventricular ejection fraction; OCT: optical coherence tomography; PCI: percutaneous coronary intervention.

Between January 2013 and January 2024, we identified 297 patients with unprotected left main disease treated by PCI in a single center in Cluj-Napoca, Romania. After applying the exclusion criteria, 237 patients were eligible for analysis; however, 16 patients were excluded from all analyses due to absence of any follow-up data. Of the 221 patients remaining in the study, 29 (13.1%) underwent OCT-guided PCI and 192 (86.9%) underwent PCI with angiographical guidance alone, without any additional imaging technique. Figure 1 shows the study composition and those excluded from this study. Baseline characteristics of the population are summarized in Table 1 and are similar between the groups in terms of age, female proportion, obesity, smoking habits, and SYNTAX score. Table 2 summarizes the procedural aspects of the enrolled population. Except for stent strut re-crossing, which was significantly higher in the OCT group (Chi square test *p* = 0.034), there were no statistically significant differences between the OCT and non-OCT groups in terms of the proportion of patients treated by a two-stent technique, final POT performed, kissing balloon inflations, or bifurcation type (true/simple) (all *p* > 0.05).

### 4.2. Primary and Secondary Endpoints

**Figure 2:**
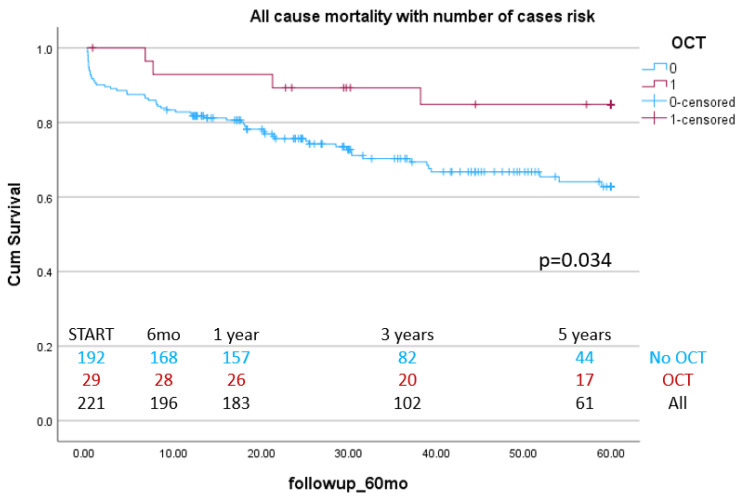
Long-term efficacy of OCT use in LM PCI: Kaplan–Meier curves for all-cause mortality between OCT- and angiography-guided PCI.

The median follow-up duration was 30.16 months (interquartile range: 14.3–60 months). Follow-up duration was limited to 60 months to ensure comparability across groups. There were 63 events overall, 4 in the OCT group and 59 in the non-OCT group. Kaplan–Meier survival analysis revealed a significantly higher survival probability in the OCT group compared to the non-OCT group (log-rank *p* = 0.034) with a mean survival time of 44.52 (95%CI 41.14–47.89) vs. 53.86 (95%CI 48.108–59.62) months (Figure 2).

Regarding the secondary endpoint, procedural success rate, there were no significant differences between the groups (98.95% vs. 100%, *p* = 0.975).

While for the non-OCT group, there were multiple variables that we were not able to obtain during follow-up, we conducted a separate analysis of the OCT group in terms of the proportion of unsatisfactory OCT results after the index procedure, leading to additional treatment, as well as TLR and MI rates at 1, 3, and 5 years (Table 3 and Table 4).

### 4.3. Multivariate Analysis

#### Multivariate Cox Regression Results

A multivariate Cox regression was conducted with 214 patients (Table 5), including 63 events and 151 censored cases, while 7 cases were excluded due to missing covariates data. Proportional hazards (PHs) were tested for each variable of interest using log-minus-log survival plots and time-dependent covariate testing. There were no significant PH violations. The analysis included follow-up time up to 60 months, and the overall model was statistically significant: (*p* = 0.003, χ^2^ (10) = 27.08,). Among the covariates, OCT—approaching significance (*p* = 0.063)—had a 63% reduction in hazard (HR = 0.370, *p* = 0.063), indicating a strong protective effect but without reaching significance; age—significant (*p* = 0.035)—with Exp (B) = 1.030, suggesting each year in age increases hazard by 3%; female sex—significant with *p* = 0.042—suggests a protective effect compared to male counterparts; and ‘true’ bifurcation type—significant with *p* = 0.027—was associated with a significant increase in mortality when compared to simple bifurcations or ostial lesions (HR = 1.787)

### 4.4. Subgroup Analysis

**Figure 3:**
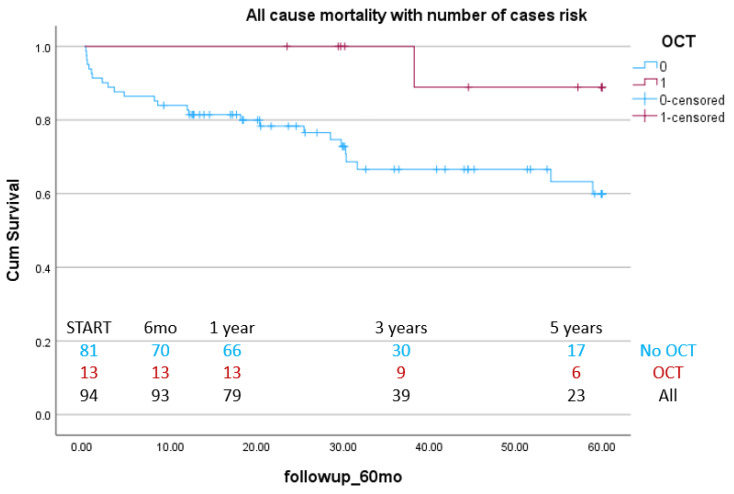
Long-term efficacy of OCT use in LM PCI: Kaplan–Meier curves for all-cause mortality between OCT- and angiography-guided PCI in elective patients.

**Figure 4:**
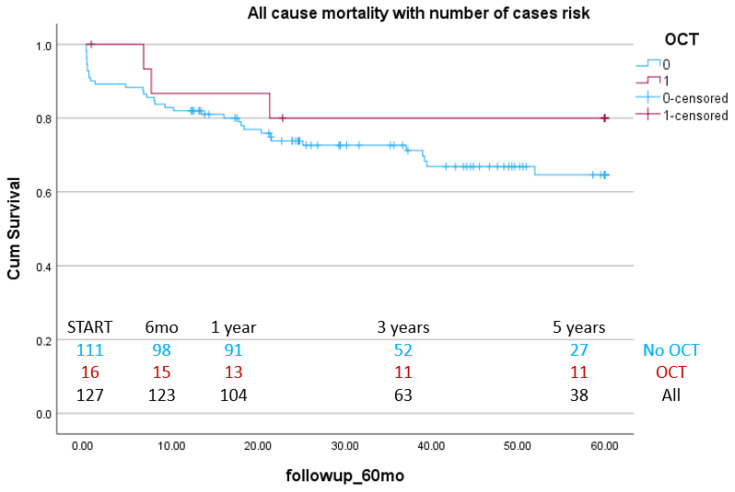
Long-term efficacy of OCT use in LM PCI: Kaplan–Meier curves for all-cause mortality between OCT- and angiography-guided PCI in NSTEMI and STEMI patients.

Additional KM survival curves were drawn for a subgroup analysis of elective coronary angiography patients and ACS patients. In both cases, the OCT group showed higher survival compared with the non-OCT group, although not reaching significance (elective subgroup log-rank *p* = 0.06, ACS subgroup log-rank *p* = 0.28) (Figure 3 and Figure 4).

## 5. Discussion

Our study aimed to investigate the impact of OCT on long-term survival rates in patients undergoing left main PCI. The main findings were as follows:

(1) OCT-guided PCI was associated with early procedural distinctions and a trend toward improved unadjusted survival in LM PCI.
(2) The multivariable Cox regression analysis did not demonstrate a statistically significant association between OCT guidance and improved long-term survival after adjusting for confounders and time-dependent effects.
(3) Subgroup analysis of elective and ACS cases showed an increase in survival for the OCT group, although not reaching significance in either subgroup.
(4) OCT guidance in LM PCI is associated with very low 5-year TLR (0.9%) and optimal MI rates (31.8%).

To begin with, in the GRAVITY registry [13], all-cause and cardiovascular (CV) deaths were the primary endpoints in 470 patients treated by PCI for LM disease. In this retrospective study, 47% of the patients died after 15 years, 17% due to CV causes. Multiple other studies, however, reported much better 10-year outcomes. In the LE MANS prospective trial, which randomly compared LM stenting and CABG for unprotected LM stenosis with low and medium SYNTAX scores, the authors reported mortality of only 30.2% in the PCI arm, with a similar probability to CABG of very long-term survival up to 14 years (74.2% vs. 67.5%; *p* = 0.34; hazard ratio: 1.45, 95% confidence interval: 0.67 to 3.13) [14]. Of note, while in the EXCEL trial, a higher rate of death from any cause and ischemia-driven revascularization in the PCI group was noted [1], the NOBLE trial reported a more frequent rate of the composite endpoint of death, non-procedural MI, stroke, and repeat revascularization in the PCI arm, with no significant differences between the two groups in terms of mortality [2,3].

While these results were encouraging, the use of intracoronary imaging has been demonstrated to improve the long-term results of complex PCI by even more. There are multiple studies that investigated the role of IVUS in left main PCI in terms of hard clinical endpoints. In the MAIN-COMPARE study, there was a significant trend towards lower mortality rates for IVUS-guided LM PCI when compared with angiography guidance (6.0% versus 13.6%, log-rank *p* = 0.063; hazard ratio, 0.54; 95% CI, 0.28 to 1.03; Cox model *p* = 0.061) [10]. Furthermore, in a metanalysis of one randomized controlled trial and nine non-randomized studies, comparing the outcomes of LM PCI with or without IVUS guidance, IVUS-guided PCI significantly reduced the risk of all-cause death compared with angiography-guided PCI (RR 0.60, 95% confidence interval [CI] 0.47–0.75, *p* < 0.001), as well as the risk of cardiac death (RR 0.47, 95% CI 0.33–0.66, *p* < 0.001), although without influencing the rates of MI and TVR between the two groups [15].

Regarding OCT, there is currently robust data regarding the impact of OCT on mortality when this imaging modality is used to guide PCI in general [16], as well as multiple studies reporting its impact on angiographic outcomes and MACE when used for guiding LM PCI [17]. However, to our knowledge, our study is the first to report on long-term mortality in OCT-guided LM PCI as compared to angiography only guidance.

The ROCK I multicentric retrospective study compared OCT guidance to IVUS or angiographic guidance for distal LM PCI, with late lumen loss (LLL) representing the primary endpoint [18]. After a median of 207 ± 23 days, LLL was significantly lower in the distal portion of the main vessel (0.03 ± 0.45 vs. 0.24 ± 0.53 mm, *p* = 025), with a similar tendency being reported for percent diameter stenosis (DS%) and restenosis rate, which were also reduced following OCT usage (14 ± 9% vs. 19 ± 16%; *p* = 0.05; 3.5% vs. 12.9%; *p* = 0.03). The authors further continued their work with the ROCK II trial, in which they compared OCT and IVUS in terms of target lesion failure (TLF) at one year, however reporting no significant differences [12], but with a higher rate of detection of acute stent malposition and residual edge dissection for OCT (10% vs. 4%; *p* = 0.04; 9.7% vs. 5.1%; *p* = 0.04).

It should also be noted that in the setting of LM disease, OCT is a more complex procedure to perform than IVUS, as it requires blood clearance, which could result in wash-out challenges, especially at the aorto-ostial segment. However, the 10-fold higher resolution of OCT over IVUS [19], makes OCT a viable alternative in the settings where detection of even minimal stent malposition is required. Furthermore, due to this higher resolution, OCT is ideal for better detection of tissue prolapse and minor dissections, and it provides better characterization of the plaque morphology, and in the setting of LM disease, these aspects might play a crucial role in obtaining a better long-term result.

In the OCTOBER trial [20] comparing imaging vs. angiographic guidance for complex bifurcation lesions, 111 patients (18.5%) in the OCT-guided PCI group and 116 (19.3%) in the angiography-guided PCI group had a bifurcation lesion involving the LM. At 2-year follow-up, the primary endpoint, represented by MACE (a composite of cardiac death, target-lesion MI, or ischemia-driven TLR) was significantly reduced by the use of OCT (10.1% vs. 14.1%, hazard ratio 0.70; 95% confidence interval, 0.50 to 0.98; *p* = 0.035). Furthermore, during the recent EuroPCR 2024, Maneveau N presented the results of a substudy of the DOCTORS trial [21,22] regarding the use of OCT in left main lesions. In that study, the use of OCT significantly reduced TLR at one year, although without exhibiting any significant improvement in the post-procedural FFR values when compared to angiography only [22]. While the main focus of these studies was not on long-term mortality, the authors scarcely reported survival information. For example, in the OCTOBER trial, cardiac death tended to be reduced by the use of OCT (1.4% vs. 2.6% (HR 0.53, 95% CI 0.22–1.25)) [20]. However, significant data on the impact of OCT guidance in LM PCI on mortality is still very limited.

In our study, the use of OCT for guiding left main procedures was associated with a higher survival probability compared to angiography-guided procedures (log-rank *p* = 0.034) at a median follow-up duration of 30.16 months. Several possible explanations could result from the OCT subgroup analysis, which showed an unsatisfactory result in almost half of the patients after the first OCT run (48%). This high rate of incomplete strut coverage, stent malposition, or underexpansion corresponds to the literature [3,17] and led to a significantly higher number of stent strut re-crossing when compared to the angiography-guided group (*p* = 0.034). However, without sufficient data for the non-OCT group, this is only a hypothesis that should be tested in randomized trials.

For a more accurate interpretation of our results, we performed a multivariable analysis of 214 patients using 10 covariates. In the overall model, OCT maintained its strong protective effect independently and had a 63% (HR = 0.37, 95% CI 0.129–1.057) reduction in all-cause death, while approaching significance (*p* = 0.06). Several independent predictors were identified to have statistical significance (age, sex, and true bifurcations), while others approached significance (LVEF-*p* = 0.067 and SYNTAX score-*p* = 0.061).

As expected, age significantly increased hazard in the multivariate model (*p* = 0.035). These findings are in accordance with those in the literature, as the IRIS-MAIN registry reported a significant treatment interaction between age ≥/< 65 years and PCI versus CABG for LM disease with respect to death, MI, and stroke, with a benefit observed for patients aged <65 years and a neutral effect for patients aged ≥65 years or older (Pint = 0.01) [23]. In the NOBLE and EXCEL registries, however, there were no interactions of age with the treatment effect on MACCE [2] or all-cause death, MI, and stroke [1,5].

In this model, female sex continued to be a significant protective factor in our group of patients, in contrast to the findings of the most important trials on LM PCI [1,2]. One possible explanation for this finding could be the small cohort of women included in our study. Strangely, furthermore, lower LVEF did not independently increase mortality over time, despite it being a well-known independent predictor in multiple studies [24,25,26]. However, in a retrospective multicenter registry of 975 patients with LM PCI, Biondi-Zoccai et al. [27] described similar findings, as in their multivariable analysis, LVEF was not an independent predictor of adverse events at any time-point. One possible explanation could be the fact that the lower the LVEF is, the more likely it is to co-occur with other unfavorable characteristics, through which it impacts the prognosis. Furthermore, it should be noted that in our study, EF was not very low (49.69 ± 8.02 vs. 50.84 ± 8.80, *p* = 0.372), which might reduce its theoretical power to independently influence mortality over a long period of time. Furthermore, of the procedural technique variables, neither final POT nor kissing balloon inflation (KBI) influenced the mortality in the multivariable analysis models. These results however, are in line with those in the literature, as POT has been described to independently influence the bifurcation-oriented composite endpoint, without significantly influencing cardiac death (*p* = 0.21) or all-cause mortality in a study of 162 patients with LM PCI [28]. Similar findings were reported for final kissing inflation as well, in a multicenter real-world registry that included 873 patients who underwent PCI with provisional stenting [29] and in which KBI did not improve long-term clinical outcomes. An increased syntax score (also approaching significance *p* = 0.061) corelated with higher hazard for all-cause mortality, similar to other literature reports, which showed that in patients with a high SYNTAX score, CABG is the preferred revascularization method, due to higher long-term mortality observed with PCI in this group of patients [1,2,4].

Lastly, bifurcation type was a significant predictor for event occurrence (*p* = 0.027, HR = 1.787, CI 95% 1.068–2.980). This indicates that ‘true bifurcation’, when compared to simple bifurcation or ostial lesions of the LM, implies a higher procedural risk. Park TK et al. also showed that true bifurcation lesions had worse clinical outcomes than non-true bifurcation lesions (HR 1.39; 95% CI 1.08–1.80; *p* = 0.01) in the COBIS II Registry, a registry of 1502 patients with true bifurcation lesions (51.8%) and 1395 with non-true bifurcation lesions (48.2%) [30], with Medina (1.1.1) and (0.1.1) being associated with a higher risk of cardiac death or MI than Medina (1.0.1) (HR 4.15; 95% CI 1.01–17.1; *p* = 0.05).

Due to the fact that in the non-OCT group, we obtained survival information from the local authorities in many cases, we were unable to identify the cause of death or secondary endpoints such as MI or TLR. However, for the patients included in the OCT group, these variables were available, and we identified a low 5-year TLR (13.6%). In addition, we performed a pre-specified subgroup analysis for the elective patients, which showed that the use of OCT was associated with higher survival probability (*p* = 0.035), in comparison to angiographic guidance, while this effect was not seen for the NSTEMI patients (log-rank *p* = 0.234). The type of clinical presentation, however, did not reach statistical significance in any of the multivariable analysis models.

There are multiple limitations which have to be taken into consideration, however. First, the retrospective nature of this observational study, spanning over a decade (2013–2024) might have limited the accuracy of the analysis. Secondly, the disparity between the group sizes (29 vs. 192) not only limits the statistical power but also amplifies the influence of outliers in the OCT group, thus influencing the multivariate model analysis by obscuring the contributions of known risk factors. Another important limitation is the difference in follow-up time between groups. OCT use was introduced earlier in the study period, resulting in longer follow-up periods for OCT patients. This temporal imbalance may artificially favor OCT in the Kaplan–Meier analysis, as survival curves extend further in this group. Efforts were made to account for this limitation through adjusted analyses and sensitivity checks. Nevertheless, even though it did not represent one of our endpoints, it would have been interesting to analyze the cause of death. However, as the data for the deceased patients was obtained from the survival registries from the local authorities, we do not have any additional information on the cause of death. Furthermore, our center is a tertiary center, therefore, the enrolled patients originate from various parts of the country, making it even harder to obtain specific follow-up information.

## 6. Conclusions

In this single high-volume center registry, the use of OCT for guiding LM PCI was associated with improved mortality in long-term follow-up. OCT showed a borderline non-significant independent effect on improving long-term mortality in the multivariable analysis. While sex, age, and bifurcation type were significant predictors and syntax score was a borderline significant predictor for long-term mortality, procedural aspects, such as POT or KBI, did not influence the outcomes. To our knowledge, our study is the first to report the effect of OCT on long-term mortality in LM PCI. However, due to the small sample size and unequal follow-up, this registry should be seen more as a hypothesis-generating study, with large randomized clinical trials needed to clearly state the long-term effect of OCT on mortality in left main PCI.

## Figures and Tables

**Table 1:** Baseline clinical characteristics.

Baseline Characteristic	Angiography-Guided Group (*n* = 192)	OCT Group (*n* = 29)	*p*-Value (Chi-Square)
Sex (Female)	55 (28.6%)	4 (13.8%)	0.087
Age	69.3 (61.7–75.6)	70.0 (60.6–74.0)	0.317
Obesity	104 (54.2%)	16 (55.2%)	0.919
Smoking	97 (50.5%)	12 (41.4%)	0.359
Diabetes Mellitus	80 (41.7%)	12 (41.4%)	0.977
Previous MI	56 (29.2%)	11 (37.9%)	0.277
Previous PCI	52 (27.1%)	9 (31%)	0.657
Atrial Fibrillation	16 (8.3%)	2 (6.9%)	0.792
STEMI	36 (18.8%)	4 (13.8%)	0.812
NSTEMI	75 (39.1%)	12 (42.4%)	0.812
Elective patients	81 (42.2%)	13 (44.8%)	0.812
LDL-C	90.88 (89.0–100.0)	100.0 (83.5–120.5)	0.098
Creatinine	1.00 (0.78–1.20)	1.00 (0.88–1.16)	0.621
LVEF	49.69 ± 8.02	50.84 ± 8.80	0.372
SYNTAX score	23.0 (20.0–25.0)	19.0 (18.0–23.0)	0.373

LDL-C: low-density lipoprotein cholesterol; LVEF: left ventricular ejection fraction; MI: myocardial infarction; NSTEMI: non-ST elevation myocardial infarction; OCT: optical coherence tomography; STEMI: ST elevation myocardial infarction.

**Table 2:** Procedural characteristics.

Variable	Angiography-Guided PCI	OCT-Guided PCI	*p*-Value (Chi-Square)
Two-stent technique	16 (8.3%)	5 (17.2%)	0.734
POT	165 (85.9%)	24 (82.8%)	0.975
POT balloon diameter	4.31 (0.67)	4.20 (1.35)	0.252
Stent diameter	4.17 (2.30)	3.94 (0.41)	0.716
Stent strut recross	44 (23.0%)	11 (42.3%)	0.034
Kissing balloon	68 (35.6%)	8 (30.8%)	0.628
Aortic protrusion	40 (21.1%)	5 (19.2%)	0.83
Additional lesions	64 (70.3%)	17 (63.0%)	0.469
Ostial lesions	37 (19.3%)	3 (10.7%)	0.54
Simple bifurcations	71 (37.0%)	11 (39.3%)	0.54
True bifurcations	84 (43.8%)	14 (50.0%)	0.54

OCT: optical coherence tomography; PCI: percutaneous coronary intervention; POT: proximal optimization technique.

**Table 3:** Long-term outcomes in the OCT-guided LM PCI group.

Variable	Number of Events in the OCT-Guided PCI Group
Additional lesion treatment after first OCT run	14 (48.27%)
1-year TLR	2 (6.9%)
3-year TLR	2 (7.4%)
5-year TLR	3 (13.6%)
1-year MI	3 (10.3%)
3-year MI	5 (18.5%)
5-year MI	7 (31.8%)
1-year all-cause death	2 (6.9%)
3-year all-cause death	2 (7.4%)
5-year all-cause death	3 (13.6%)

MI: myocardial infarction; OCT: optical coherence tomography; TLR: target lesion revascularization.

**Table 4:** OCT group sub-analysis.

Variable	Number of Patients	*n* (%)
Two stent technique	29	6 (20.6%)
Additional lesion present	29	18 (62.0%)
Additional lesion treated (regardless of OCT)	18	12 (66.6.0%)
OCT re-check performed	29	25 (86.2%)
OCT result unsatisfactory	25	12 (48.0%)
Additional treatment after OCT	25	12 (48.0%)
Final OCT result satisfactory	29	29 (100.0%)

**Table 5:** Variables used in multivariate analysis.

Variable	*p*-Value	HR [Exp (B)]	95% CI for HR
AGE	0.035	1.03	[1.002–1.058]
SEX	0.042	0.507	[0.264–0.975]
CREATININ	0.921	1.024	[0.638–1.644]
LVEF	0.067	1.036	[0.988–1.076]
INDICATION (ACS vs. elective)	0.899	1.038	[0.614–1.756]
POT yes/no	0.604	1.223	[0.517–2.619]
KISSING	0.42	0.789	[0.443–1.403]
OCT	0.063	0.37	[0.129–1.057]
SYNTAX score	0.061	1.037	[0.998–1.076]
Bifurcation Type ‘true’ vs. simple/ostial	0.027	1.787	[1.068–2.990]

## Data Availability

The original contributions presented in this study are included in this article. Further inquiries can be directed to the corresponding author.
